# Predictive Value of the Respiratory Variation in Inferior Vena Cava Diameter for Ventilated Children With Septic Shock

**DOI:** 10.3389/fped.2022.895651

**Published:** 2022-07-07

**Authors:** Zihong Xiong, Guoying Zhang, Qin Zhou, Bing Lu, Xuemei Zheng, Mengjun Wu, Yi Qu

**Affiliations:** ^1^Department of Pediatrics/Key Laboratory of Birth Defects and Related Diseases of Women and Children (Ministry of Education), West China Second University Hospital, Sichuan University, Chengdu, China; ^2^Department of Pediatric Intensive Care Unit, Chengdu Women's and Children's Central Hospital, School of Medicine, University of Electronic Science and Technology of China, Chengdu, China; ^3^School of Medicine, University of Electronic Science and Technology of China, Chengdu, China; ^4^Department of Anesthesiology, Chengdu Women's and Children's Central Hospital, School of Medicine, University of Electronic Science and Technology of China, Chengdu, China

**Keywords:** echocardiography, fluid responsiveness, hemodynamic, inferior vena cava, sepsis shock, children

## Abstract

**Objectives:**

This study aimed to investigate the predictive utility of respiratory variations of inferior vena cava diameters on fluid responsiveness in children with septic shock.

**Design:**

A prospective observational single-center study.

**Setting:**

A pediatric intensive care unit in a tertiary hospital in China.

**Participants:**

Patients with sepsis shock who require invasive mechanical ventilation were recruited between 1 December 2017 and 1 November 2021.

**Interventions and Measurements:**

Volume expansion (VE) was induced by a 30-min infusion of 20 ml/kg of normal saline. Hemodynamics indexes were obtained through bedside transthoracic echocardiography (TTE) measurement and calculation.

**Results:**

A total of 86 patients were enrolled in this study, among them, 45 patients (52.3%) were considered to be non-responders (NR), with an increase in stroke volume variation (SVV) <15% after VE. Multivariate logistic analysis showed that ΔIVC (adjusted OR = 1.615, 95% CI 1.092–2.215, *p* = 0.012) was the significant predictor associated with the fluid responsiveness. The area under the ROC of ΔIVC was 0.922 (95% CI: 0.829–1.000, *p* < 0.01), and the cutoff value of ΔIVC used to predict fluid responsiveness was 28.5%, with a sensitivity and specificity of 95.4 and 68.5%, respectively.

**Conclusions:**

The ΔIVC was found to have a potential value in predicting fluid responsiveness in mechanically ventilated children with septic shock.

## Introduction

Sepsis shock is a leading cause of death in children globally. The mortality rate of septic shock is as high as 25%, despite advanced supportive care (1). Currently, fluids therapy remains the cornerstone of the hemodynamic resuscitation of children with septic shock. Fluid resuscitation seeks to rapidly restore the effective circulating blood volume and oxygen delivery to organs. Effective fluid resuscitation can improve the prognosis (2, 3), while repeated or inappropriate fluid bolus administration is associated with increased mortality and the length of intensive care unit (ICU) stay (4). Previous studies have shown that only 40–69% of children respond to intravascular volume expansion (5–8)_._ Fluid overload is a common complication during the resuscitation of septic shock and can be avoided by predicting fluid responsiveness. Therefore, we have challenges in identifying which patients will respond to volume expansion.

Numerous hemodynamic variables could be predictors of fluid responsiveness (9, 10). Traditional static pressure indexes such as central venous pressure (CVP) or mean arterial pressure (MAP) had a limited predictive value for fluid resuscitation (11, 12). Dynamic indices have shown to be good predictors of fluid responsiveness in recent years, especially the indices relying on heart-lung interactions, such as inferior vena cava respiratory variability index, velocity-time integral (VTI) in left ventricular inflow tract, stroke volume variation (SVV), and so on (13–15). VTI or SVV was able to reliably predict the fluid loading response and was superior to traditional pressure indexes for adults (16). Children may have different patterns of fluid responsiveness under critical care conditions. The predictive value of these dynamic variables remains unclear (5, 17). Apart from this, VTI and SVV monitoring need highly skilled operators to ensure measurement accuracy and require specialized pieces of equipment that are not available in every hospital. These make them more difficult to perform in the clinical environment. Thus, more ICU physicians are willing to choose the inferior vena cava (IVC) as a point-of-care parameter of volume status assessment due to their convenience for measuring. A systematic review and meta-analysis found respiratory variations in the inferior vena cava diameter performed moderately well in predicting fluid responsiveness in adult patients with circulatory shock receiving mechanical ventilation (18). However, the value of IVC in predicting fluid responsiveness among pediatric populations was not clear.

Thus, we performed the single-center prospective observational study to evaluate the predictive utility of respiratory changes in IVC diameter for fluid responsiveness in children with septic shock undergoing mechanical ventilation as well as to provide evidence-based clues for clinically reasonable fluid therapy in these patients.

## Methods

### Research Population

The patients with septic shock from 1 December 2017 to 1 November 2021 were recruited for this study. All parents or guardians of the patients voluntarily gave written informed consent before the trial. Patients did not receive any stipend for participation. This study was approved by the Chengdu Women's and Children's Central Hospital in Sichuan, China [Registration No. 2017(11)].

The inclusion criteria were as follows: (1) children aged 6 months to 5 years; (2) patients who fulfilled the criteria for septic shock according to the Surviving Sepsis Campaign (19). Septic shock in children is defined as a severe infection leading to cardiovascular dysfunction, including hypotension, need for the vasoactive drug, or impaired tissue perfusion (such as deterioration of mental status, tachycardia, prolonged capillary refill of >2 s, mottled and clammy skin, decreased urine output, or elevated blood lactate, and so on); only community-acquired septic shock cases and patients who had not received previous fluid boluses before entry into the study were included. (3) Patients with septic shock who underwent invasive mechanical ventilation and required rapid fluid resuscitation at the discretion of the pediatric intensive care unit (PICU) physicians.

The exclusion criteria were as follows: children with contraindications to rehydration testing (acute coronary syndrome, cardiogenic shock, or severe pulmonary edema), cardiac arrhythmias, right ventricular dysfunction, tricuspid regurgitation, heart failure, intra-abdominal hypertension, contraindications to central venous or artery line placement, congenital heart diseases, patients with strong spontaneous respiratory effort or informed consent not obtained from parents or guardians.

### Monitoring and Treatment

Eligible patients were monitored for their heart rate (HR), ambulatory blood pressure (BP), a five-lead electrocardiogram, and transcutaneous oxygen saturation. A radial arterial line was placed to continuously monitor invasive arterial BP and MAP. Meanwhile, patients underwent right internal jugular vein catheterization, with the catheter tip being located at the opening of the right atrium, and the position was confirmed with bedside radiography. CVP was measured *via* jugular central venous catheters at end-expiration. The patients' clinical information and laboratory results regarding capillary refill time, Ramsay sedation score, arterial blood gas analysis, lactate, origin of sepsis and ventilator parameters, and Pediatric Risk of Mortality (PRISM) score (20) were obtained from the electronic medical record.

All patients were given adequate midazolam and remifentanil sedation maintaining a Ramsay score of 5–6 (21, 22). Vecuronium may be considered when the patients have a strong work of the respiratory muscle. The ventilator (Maquet, Servo-s, Germany) was set with Assist/Control mode. The ventilator parameters included positive end-expiratory pressure (PEEP) 3-6 cmH_2_O, the inspiratory-to-expiratory 1:2, and tidal volume (VT) 8-10 ml/kg (18, 23). The ventilatory settings were kept constant throughout the study. Moreover, antimicrobial therapy, source control, and organ support were following the clinical guidelines and other accepted expert consensus. Patients had not received any vasoactive agents during the study.

### Volume Expansion (VE)

A rapid intravenous infusion (20 ml/kg of 0.9% normal saline) was performed within 30 min. We used the change of SV after rapid volume infusion as the criterion for judging volume responsiveness in our study. SVI (%) = (SV_afterVE_-SV_beforerVE_)/ SV_beforerVE_ X 100%. Patients with SVI >15% from baseline were classified as the responders (R), and those with SVI <15%, unchanged or even decreased, were considered the non-responders (NR) (10).

### Echocardiographic Measurements

**Figure 1:**
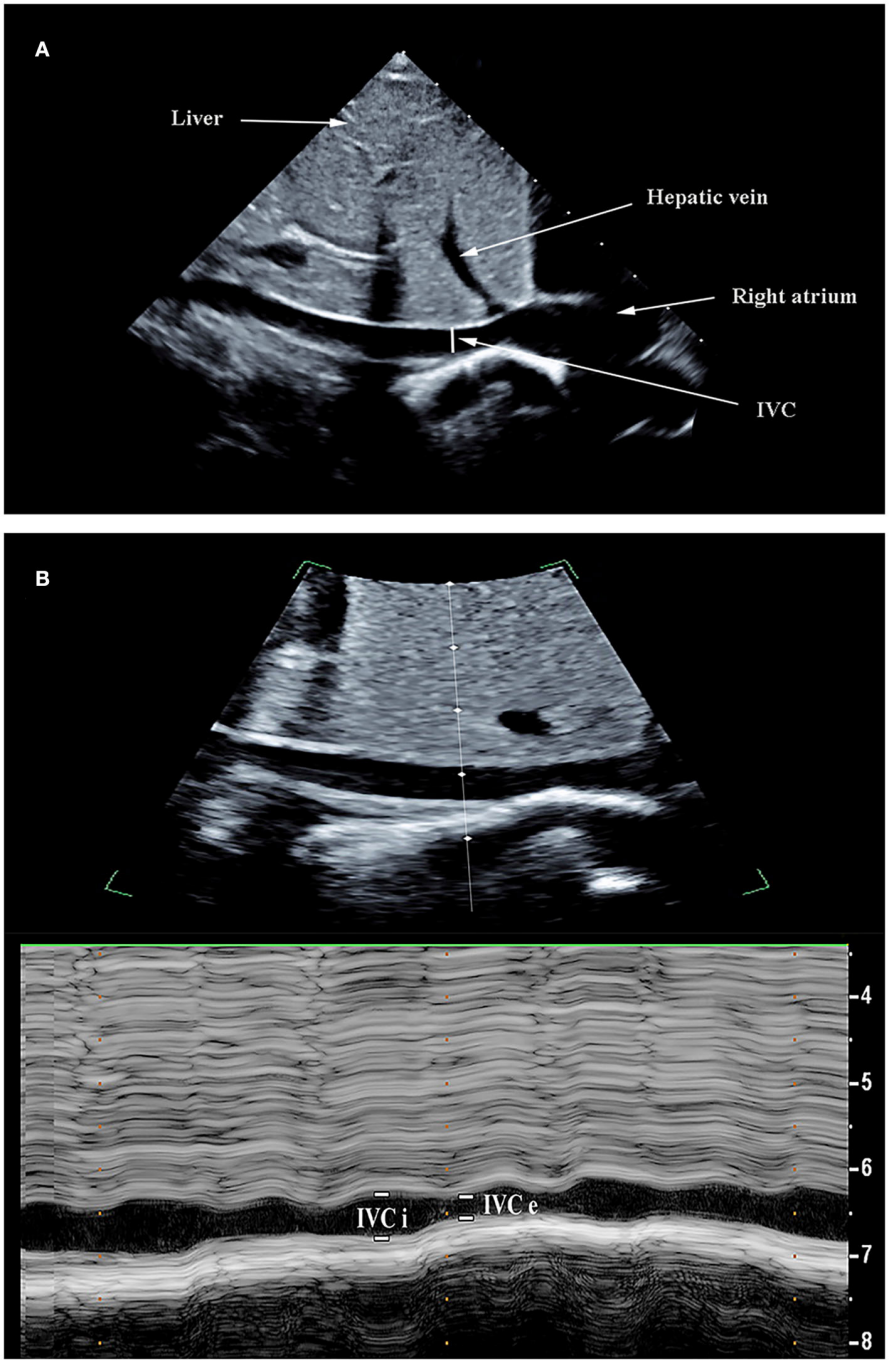
Ultrasonographic measurement of inferior vena cava (IVC) diameters. The IVC images were obtained using the bi-dimensional mode on a subcostal long-axis view **(A)**. M-mode line was placed through the IVC 1–2 cm caudal from the hepatic vein-IVC confluence and an M-mode tracing obtained. The IVC maximum diameter (IVCmax) and IVC diameter (IVCmin) was measured during the respiratory cycle **(B)**.

Transthoracic echocardiography (TTE) examination was performed using a bedside ultrasound device (Philips Ultrasound CX50, the Netherlands) equipped with a phased array transthoracic probe (S5-1). All patients remained in a supine position during the execution of the study protocol. Hemodynamics indexes were obtained on the same child at two time points, before VE and after VE. The IVC diameter was measured from the subcostal longitudinal plane when the M-mode tracing was exactly perpendicular to the IVC and 1 cm distal to the hepatic vein-IVC confluence (24, 25). The maximal diameter of the IVC (IVCmax) and the minimal diameter of the IVC (IVCmin) was obtained within a measured respiratory cycle (Figure 1). The inferior vena cava variability (ΔIVC) was calculated as the following equation: ΔIVC = (IVCmax-IVCmin) / [(IVCmax+IVCmin)/2] x100 (%). The distensibility index of the inferior vena cava (dIVC) was calculated as follows: dIVC = (IVCmax-IVCmin) /IVCmin x100 (%). The measurements above were repeated three times during three consecutive respiratory cycles and the average values were taken for statistical analysis. VTI was measured from an apical five-chamber view by using a pulsed-wave Doppler. VTI measurements were performed on at least 6 consecutive waveforms and the mean value was obtained (26). The left ventricular ejection fraction (EF) was measured from the parasternal short axis (M-mode). The aortic diameter (D) was measured from the parasternal long-axis view at the level of the aortic annulus. The left ventricular stroke volume (SV) was obtained using the following equations: SV = VTI x π(D2)/4. SVV_tte_ has previously been shown to have the diagnostic value for predicting fluid responsiveness in children under mechanical ventilation (27). SVV_tte_ was the variation of SV during the ventilatory cycle. The calculation formula is as follows: SVV_tte_ =(SVmax-SVmin)/[(SVmax+SVmin)/ 2]x100 (%). Each measurement period was at least 20-s long. The mean of the three measurements was used for statistical analysis. To reduce interobserver variability, all ultrasound operations were conducted by the same operator (Qin Zhou, who holds a certification in ultrasound evaluation, has an 11-year experience in PICU), and was blinded to the hemodynamic variables collected by other investigators (Bing Lu and Zi-Hong Xiong). The recorded ultrasound images were later reviewed by sonographers (within 24 h) to ensure that high-quality image acquisition and accurate interpretations are being performed.

### Statistical Analysis

All statistical analyses were performed using SPSS 20.0 (IBM Corp, Armonk, NY, United States). The normalities of data distributions were confirmed using the Kolmogorov–Smirnov test. The normally distributed quantitative data were presented as the mean ± standard deviation (mean ± SD). The categorical variables were presented as frequencies and proportions. Abnormally distributed continuous variables were presented as the median (25th and 75th percentiles). The comparisons of variables before and after volume expansion using the paired *t*-test or Friedman test were conducted. Qualitative data were compared by the X^2^ test. Multivariable logistic regression analyses were performed to determine the predictive factors of fluid responsiveness. Odds ratios (ORs) with 95% confidence intervals (CIs) were calculated. Receiver operating characteristic (ROC) curves were established to assess the predictive utility of variables for fluid responsiveness. The optimal cutoff value based on the ROC curve was defined as the maximized value for the sum of sensitivity and specificity. Statistical significance was set at *p* <0.05.

## Results

### Demographics and Clinical Characteristics

A total of 86 mechanically ventilated patients with septic shock were included during the study period. Among them, 41 patients were classified into the R group and 45 patients into the NR group. The non-response rate of the total included participants was 52.3%. The non-responders had longer lengths of stay and greater mortality compared with responders. There was no significant difference in the demographic data (age, gender, weight, body surface area) or clinical characteristics (pediatric risk of mortality score, Ramsay score, inhaled oxygen concentrations, ventilatory parameters, capillary refill time, lactate, arterial pH, arterial PaO_2_) between the R group and NR group. The baseline demographics and clinical characteristics are described in Table 1.

**Table 1:** Baseline clinical characteristics and comparison between responders and non-responders (before volume expansion) quantitative data was shown as ($\bar{x}$ ± SD) or median (25th and 75th percentiles).

**Characteristics**	**Responders (n = 41)**	**Non-responders** **(*n* = 45)**	***P*-value**
Age (years)	1.7 ± 0.8	2.8 ± 1.2	0.316
Gender M/F (n)	20 / 21	28 / 17	0.507
Weight (Kg)	11.5 ± 1.6	13.2 ± 2.7	0.370
BSA (m^2^)	0.50 ± 0.06	0.56 ± 0.05	0.352
PRISM III score	8.2 (7.8, 9.2)	9.1 (8.4, 9.6)	0.012
Ramsay score	5.2 (5, 6)	5.5 (5, 6)	0.471
LOS in PICU, days	7.6 (3, 10)	13.5 (7, 19.5)	0.004*
PICU Mortality , n(%)	2 (4.9%)	7(15.6%)	0.001*
Inhaled oxygen concentrations(%)	32%	38%	0.088
Ventilatory parameters			
PEEP (cmH_2_O)	4.2 (4.0, 5.0)	4.6 (4.0, 5.0)	0.075
Pplat (cmH_2_O)	19 (14, 26)	20 (15, 27)	0.462
Exhaled tidal volume (ml/kg ideal body weight)	6.7 (6.0, 8.0)	6.9 (6.0, 8.0)	0.089
Origin of sepsis, n			
Pneumonia	18	22	
Gastrointestinal sepsis	9	9	
Meningtitis	6	5	
Skin or urinary	4	4	
Septicemia without focus	3	5	
Capillary refill time(s)	3 ± 1	3 ± 1	0.560
Lactate	2.8 (1.5-3.9)	2.9 (1.5-4.2)	0.663
Arterial pH	7.36 ± 0.15	7.34 ± 0.12	0.682
Arterial PaO_2_	74 (43-90)	66 (42-87)	0.023

Qualitative data was showed as numbers. BSA, body surface area; PCIS, pediatric critical illness score; LOS: length of stay; PRISM, pediatric risk of mortality; PEEP, positive end-expiratory pressure; Pplat, plateau pressure; PaO2, partial pressure of arterial arterial oxygen.^*^P < 0.05.

### Comparison of Hemodynamic Parameters

Hemodynamics data and echocardiographic measurements in the R group and NR group before and after VE are shown in Table 2. There were significant differences in hemodynamic parameters (HR, CVP, SVV, ΔIVC, and dIVC)before volume expansion between the R and NR groups (*p* < 0.05). Volume expansion significantly increased the mean EF from 53.64 to 57.09 and the mean MAP from 52.72 to 60.44 in R (*p* < 0.05), while there was a significant increase of CVP in both the R and NR groups (*p* < 0.05). HR, SVV, ΔIVC, and dIVC decreased significantly after volume expansion in the R group (*p* < 0.05 for all comparisons).

**Table 2:** Comparison of hemodynamic parameters before and after volume expansion.

**Variables**	**Before VE**	**After VE**	***P* intragroup**
HR (beatsmin^−1^)			
Responders	159 ± 17	139 ± 15	0.000*
Non-Responders	143 ± 14	141 ± 13	0.306
*P* intergroup	0.017*	0.216	
MAP (mmHg)			
Responders	52 (48, 57)	60 (58,63)	0.002*
Non-Responders	54 (48, 59)	58 (49, 66)	0.071
*P* intergroup	0.682	0.009	
CVP (cmH2O)			
Responders	4.1 ±1.7	7.0 ± 1.2	0.000*
Non-Responders	7.7 ±1.6	9.9 ± 2.5	0.000*
*P* intergroup	0.000 *	0.000*	
EF (%)			
Responders	53.6 ± 3.8	57.1 ± 1.6	0.000*
Non-Responders	51.8 ± 4.7	52.8 ± 4.9	0.682
*P* intergroup	0.075	0.000 ^*^	
SVVtte (%)			
Responders	18.2 ± 5.2	11.3 ± 3.5	0.000*
Non-Responders	10.2 ± 1.4	9.7 ± 2.0	0.307
*P* intergroup	0.000 *	0.103	
ΔIVC (%)			
Responders	26.0 ± 4.2	16.1 ± 5.1	0.000*
Non-Responders	17.3 ± 6.7	16.4 ± 5.6	0.071
*P* intergroup	0.000*	0.843	
dIVC (%)			
Responders	29.1 ± 5.0	20.9 ± 5.3	0.008 *
Non-Responders	18.5 ± 6.6	17.6 ± 5.1	0.091
*P* intergroup	0.000*	0.062	

*Data are shown as ($\bar{x}$ ± SD) or median (25th and 75th percentiles).*

*HR, heart rate; MAP, mean blood pressure; CVP, central venous pressure; SVV, stroke volume variation; TTE, transthoracic echocardiography; EF, ejection fraction; ΔIVC, the inferior vena cava variability; dIVC, distensibility index of the inferior vena cava.*

^*^P < 0.05.

### Association of Hemodynamic Variables With Fluid Responsiveness

Variables with statistically significant differences (*p* < 0.05) between the R group and the NR group were tested in multivariate logistic analysis. The results showed that ΔIVC (OR = 1.615, 95% CI 1.092–2.215, *p* = 0.012) was a significant predictor associated with the volume responsiveness when it was adjusted for MAP, CVP, and HR. MAP (OR = 0.862, 95% CI 0.733–0.975, *p* = 0.03), and CVP (OR = 4.492, 95% CI 1.261–15.964, *p* = 0.02) were also independent predictors. No significant differences were found for HR and dIVC (Table 3).

**Table 3:** Multivariate analysis of the predictors for volume responsiveness.

**Variables**	**Adjusted OR (95% CI)**	***P*-value**
ΔIVC	1.615 (1.092, 2.215)	0.012*
HR	1.070 (0.985, 1.159)	0.170
MAP	0.862 (0.733, 0.975)	0.030*
CVP	4.465 (1.261, 15.964)	0.020*
dIVC	1.069 (0.985, 1.159)	0.113
HR	1.069 (0.946, 1.207)	0.287
MAP	0.857 (0.745, 0.986)	0.031*
CVP	4.492 (1.263, 15.976)	0.020*

HR, heart rate; MAP, mean blood pressure; CVP, central venous pressure; ΔIVC, the inferior vena cava variability; dIVC, distensibility index of the inferior vena cava. ^*^p < 0.05.

### Comparison of ΔIVC, MAP, and CVP as Predictors of Fluid Responsiveness

**Figure 2:**
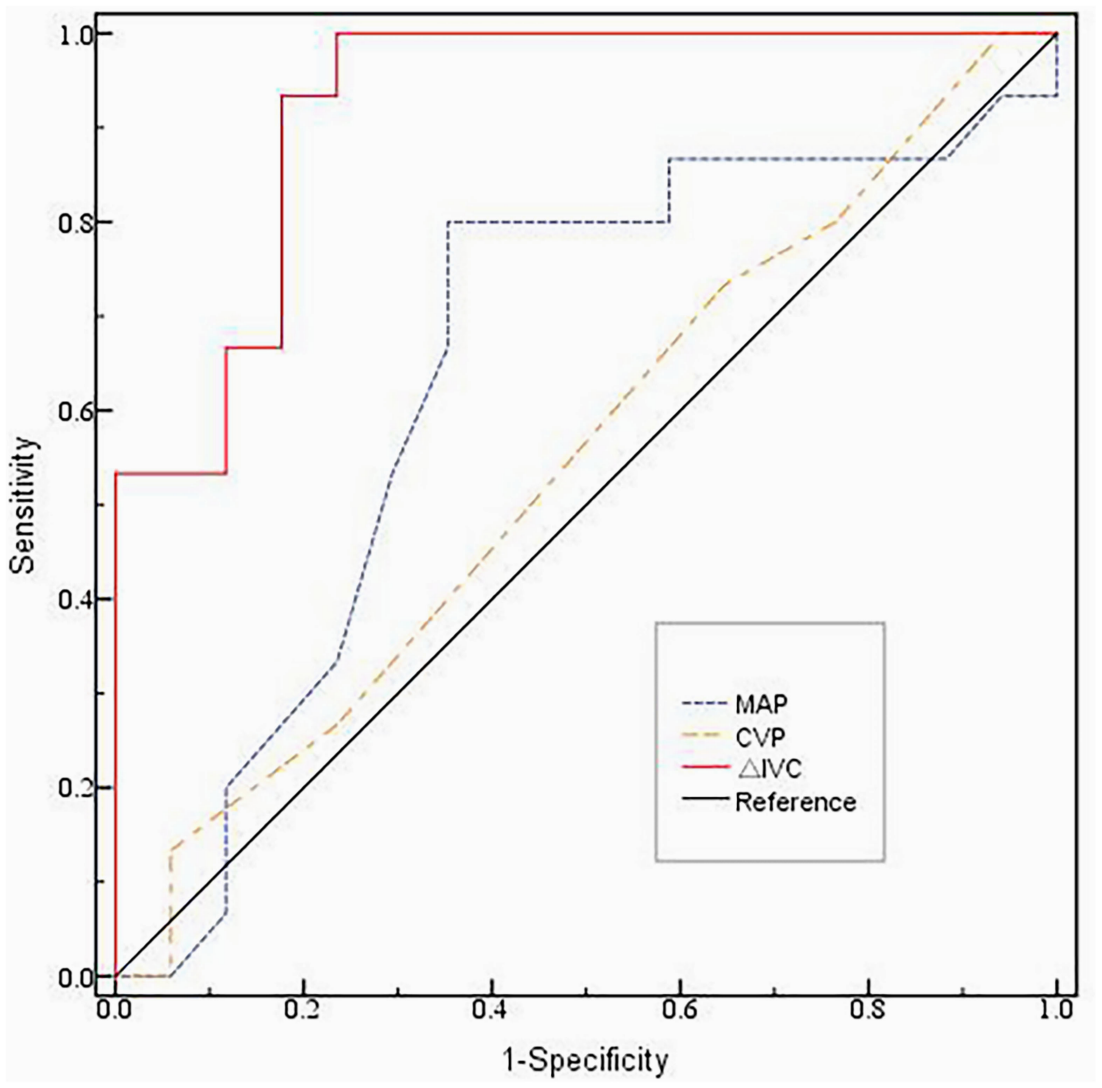
The ROC curve of the ΔIVC, MAP and CVP before volume expansion.The area under the ROC (AUROC) of ΔIVC was 0.922 (95% CI: 0.829–1.000, *p* < 0.01). The AUROC of MAP was 0.645 (95% CI: 0.444–0.847, *p* = 0.162). CVP had an AUROC of 0.549 (95% CI: 0.347–0.751, *p* = 0.637). The cutoff value of ΔIVC used to predict fluid responsiveness was 28.5%, with a sensitivity and specificity of 95.4% and 68.5%, respectively. ROC, Receiver Operating Characteristic; MAP, mean blood pressure; CVP, central venous pressure; ΔIVC, the inferior vena cava variability.

The indictors (ΔIVC, MAP, CVP) used to compare were from the R group before volume expansion. The results of the receiver operating characteristic (ROC) curve analysis are provided in Figure 2. The area under the ROC (AUROC) of ΔIVC was 0.922 (95% CI: 0.829–1.000, *p* < 0.01), and the cutoff value of ΔIVC used to predict fluid responsiveness was 28.5%, with a sensitivity and specificity of 95.4 and 68.5%, respectively. The AUROC of MAP was 0.645 (95% CI: 0.444–0.847, *p* = 0.162). CVP had an AUROC of 0.549 (95% CI: 0.347–0.751, *p* = 0.637).

## Discussion

This study was undertaken to identify the respiratory variations in IVC diameter as a predictor of fluid responsiveness. Our results show that ΔIVC at a cutoff value of 28.5% can be used to predict fluid responsiveness in mechanically ventilated children with septic shock. It appeared that the ΔIVC was more effective in the prediction of the fluid responsiveness when compared to the other indicators, such as dIVC, CVP, MAP, and HR.

Currently, two major approaches are being used for assessing fluid responsiveness: the static and dynamic approaches. In this study, we found a significant increase in CVP in both R and NR groups after volume expansion (*p* < 0.01). Elevated CVP may be related to intravenous fluids treatment before admission. The area under the ROC of CVP was only 0.549. The result showed that CVP had no significant diagnostic value for predicting fluid responsiveness. This is consistent with previous reports that CVP, which is a classical static hemodynamic variable, cannot reliably predict fluid responsiveness (12, 28). A meta-analysis study (29) also confirmed that CVP can be regarded as an indicator of right ventricular end-diastolic volume index and can therefore not be regarded as an indicator of preload responsiveness to guide fluid therapy. Meanwhile, MAP did not indicate a significant predictive value in evaluating fluid responsiveness in our study. After volume expansion, the different amplitude of elevations in MAP was seen in both groups. Blood pressure is mainly determined by stroke volume and arterial elastance. The magnitude of the BP elevation caused by an increase in stroke volume may be partially offset due to a lower arterial elastance in children. Although these changes in MAP in fluid responders were statistically, MAP is still not a reliable indicator of predicting volume.

The most important treatment strategy for patients with septic shock is initial fluid resuscitation to restore hemodynamic stability. According to the Frank-Starling principle, increasing the heart preload can significantly improve the cardiac output only when both the left and right ventricles are in the elevated phase of cardiac function. It has been unclear as to which patients are volume-responsive and likely to benefit from fluid resuscitation. Although fluid resuscitation therapy is crucial in the management of hemodynamically unstable critically ill patients, excessive or inadequate fluid resuscitation can be harmful. A similar finding was shown in our study. Of the 58 patients with septic shock, 31 patients (53.4%) were non-responsive to volume expansion. In the NR group, it did not show a significant increase in cardiac output or a significant improvement in the heart rate and blood pressure after volume expansion. Judging the patients' volume status remains challenging.

Bedside ultrasound is an important method of evaluating the volume status of critically ill patients and has been widely adopted in intensive care units. Moreover, point-of-care ultrasound is a convenient and non-invasive method to perform hemodynamics measurements and hemodynamic monitoring (30). This hemodynamic information would be useful for the implementation of goal-directed hemodynamic therapy and help to tailor better fluid therapy protocol. Compared with other echocardiographic measurements, such as SV, VTI, and aortic peak blood flow velocity variation rate of breathing, the measurement of IVC diameter is relatively easy. The cardiac output may induce cyclic changes in intrathoracic pressure, especially in patients undergoing mechanical ventilation under deep sedation. Hence, there is a significant increase in the percentage of IVC diameter change in patients with volume deficits. Some studies (17, 31) suggest that respiratory change in IVC diameter is a useful predictor of fluid responsiveness. However, a systematic review and meta-analysis (32) stressed that ultrasound evaluation of the diameter of the IVC and its respiratory variations in fluid responsiveness had extreme heterogeneity. These results were inconsistent. Several factors affect the accuracy of the IVC for volume status judgments: the measurement sites of inferior vena cava diameter (33), the skill of the operators, the heart function, abnormal intrathoracic pressure, intra-abdominal hypertension, and so on.

Therefore, more stringent inclusion and exclusion criteria were developed in our study. We enrolled children with septic shock who received controlled mechanical ventilation under sedation and analgesia to extensively eliminate the effects of voluntary breathing and improve mechanical synchronism. Moreover, we included patients with moderate PEEP levels and a tidal volume ≤ 10 ml/kg to minimize the effects of PPEP and intrathoracic pressure on the diameters of IVC. Eventually, we found that the cutoff value of ΔIVC (before volume expansion) for predicting fluid responsiveness was 28.5%. Kutty et al. (34) reported that IVC collapsibility varied with body surface area (BSA), and the average was above 30% in normal pediatric subjects since this average value is higher than the 28.5% cutoff in this study. The interpretation of the inconsistent results can consider the following factors: intrathoracic pressure changes were highly regulated and the influence of spontaneous breathing is maximally removed. Furthermore, we found that the sensitivity of a ΔIVC > 28.5% to predict fluid responsiveness was 0.954 with a specificity of 0.685. While the sensitivity of ΔIVC was good, the specificity was not high. A voluntary inspiratory effort may explain in part the relatively low specificity of ΔIVC. The respiratory variation of the IVC diameter will increase accompanied by strong spontaneous breathing. However, we were not able to confirm whether fully eliminating the work of breathing and the spontaneous breathing efforts had no influence on the respiratory variation of the IVC diameter in our study. In addition„ other factors could also affect the relatively low specificity of ΔIVC: intrathoracic pressure changes, CVP, lung compliance, airway resistance, and so on (35, 36).

Moreover, in this study, we found that dIVC was not the significant predictor associated with volume responsiveness. Saritaş et al.'s (37) study showed the dIVC had a more accurate predictive role in predicting the volume status when compared with the ΔIVC among the patients with spontaneous respiration receiving different positive pressure support. Another research (38) showed the IVC area distensibility index and its diameter ratio in cross-section had more value than the IVC diameter distensibility index for predicting fluid responsiveness in mechanically ventilated patients. Thus, it still needs further research to confirm the value of dIVC.

This study found that the ΔIVC was a useful predictor with the ability to predict responsiveness in mechanically ventilated children with septic shock. The result was obtained in a selected patient population treated by specific protocols. The not so high specificity of ΔIVC suggests that clinicians should have a comprehensive evaluation of the ΔIVC in conjunction with the patient's underlying disease status and other hemodynamic changes, thereby, contributing to clinical fluid management.

## Data Availability Statement

The raw data supporting the conclusions of this article will be made available by the authors, without undue reservation.

## Ethics Statement

The studies involving human participants were reviewed and approved by the Chengdu Women's and Children's Central Hospital in Sichuan, China [Registration No. 2017(11)]. Written informed consent to participate in this study was provided by the participants' legal guardian/next of kin.

## Author Contributions

Study design and Project administration: GZ and YQ. Acquisition of data: ZX, QZ, GZ, and BL. Formal analysis: ZX and MW. Writing original draft: ZX and XZ. Writing review and editing: ZX, YQ, and MW. Funding acquisition: YQ. All authors have read and agreed to the published version of the manuscript.

## Funding

This work was supported by the National Natural Science Foundation of China (81771634 and 81971428) and the Fundamental Research Funds for the Central University (SCU2020D006).

## Conflict of Interest

The authors declare that the research was conducted in the absence of any commercial or financial relationships that could be construed as a potential conflict of interest.

## Publisher's Note

All claims expressed in this article are solely those of the authors and do not necessarily represent those of their affiliated organizations, or those of the publisher, the editors and the reviewers. Any product that may be evaluated in this article, or claim that may be made by its manufacturer, is not guaranteed or endorsed by the publisher.
